# The role of exhaust ventilation systems in reducing occupational exposure to organic solvents in a paint manufacturing factory

**DOI:** 10.4103/0019-5278.43266

**Published:** 2008-08

**Authors:** Mohammad Javad Jafari, Ali Karimi, Mansoor Rezazadeh Azari

**Affiliations:** Occupational Health Department, Faculty of Health, University of Shahid Beheshti (MC), Tehran, Iran; 1 Occupational Health Department, Faculty of Health, University of Tehran, Tehran, Iran; 2 Occupational Health Department, Faculty of Health, University of Shahid Beheshti (MC), Tehran, Iran

**Keywords:** Exhaust ventilation systems, occupational exposure, paint manufacturing, ventilation standard

## Abstract

This paper presents the successful design and implementation of several exhaust ventilation systems in a paint manufacturing factory. The ventilation systems were designed based on American Conference of Governmental Industrial Hygienists recommendations. The duct works, fans, and other parts were made and mounted by local manufacturers. The concentrations of toluene and xylene as the common solvents used in paint mixing factories were measured to evaluate the role of ventilation systems in controlling the organic solvents. Occupational exposure to toluene and xylene as the major pollutants was assessed with and without applying ventilation systems. For this purpose, samples were taken from breathing zone of exposed workers using personal samples. The samples were analyzed using Occupational Safety and Health Administration analytical method No.12. The samples were quantified using gas chromatography. The results showed that the ventilation systems successfully controlled toluene and xylene vapors in workplace, air well below the recommended threshold limit value of Iran (44.49 and 97.73 ppm, respectively). It was also discovered that benzene concentration in workplace air was higher than its allowable concentrations. This could be from solvents impurities that require more investigations.

## INTRODUCTION

Many industrial sectors use organic solvents widely.[1] Nervous system damage (central and peripheral), kidney, and liver damage, adverse reproductive effects, such as sperm changes and infertility, skin lesions, and cancer, are the major health impacts associated with organic solvent exposure.[2] They can also cause death from acute exposure, leading to depression of the brain's respiratory center and/or cardiac arrhythmias. Solvents share many chemical, physical, and biological properties, which warrant that national attentions need to be directed to them as a group. In addition, many solvent groups or individual substances have special properties that require specific control measures.

Solvents are one of the most important components of paint and have the major purpose of reducing (thinning) paints to a suitable handling consistency or viscosity for ease of manufacture and application. After the paint has been applied, the solvent evaporates and leaves the dry paint film on the substrate. In paint production, solvents vapors are emitted throughout the manufacturing process. If these emissions are left uncontrolled, high concentrations of organic solvents can build up in the work area, compromising workers’ health and safety. Release of volatile organic solvents to the atmosphere can result in increased levels of tropospheric ozone, a pollutant that causes negative health effects in the human pulmonary system.[3] Modification of equipment and process, improvement of operating practices, and recycling may lead to lower organic solvent emissions from the process. The application of appropriate exhaust ventilation is used to remove the contaminants generated by an operation to maintain a healthful work environment.[4] For this purpose, well-designed capturing (hoods), removing (air cleaning) devices and appropriate execution of ductworks are significant.[4]

Paints are usually a mixture of 45% low solvent-based, 45% high solvent-based paints, and 10% thinners. The primary factors affecting emissions from paint manufacturing process are the types of solvents used and mixing temperature. McMinn believe that even in well-controlled conditions, about 1-3% of the solvent is lost in paint manufacturing process.[4] The emission rate for the uncontrolled manufacturing of paints and varnishes is 15 g/kg of consumed solvent. The main purpose of present study was to determine the capabilities of implementing well-known ventilation standards (VS) in controlling indoor air pollutions from solvents used in a paint manufacturing factory.

## MATERIALS AND METHODS

### Ventilation system design and test

**Figure 1:**
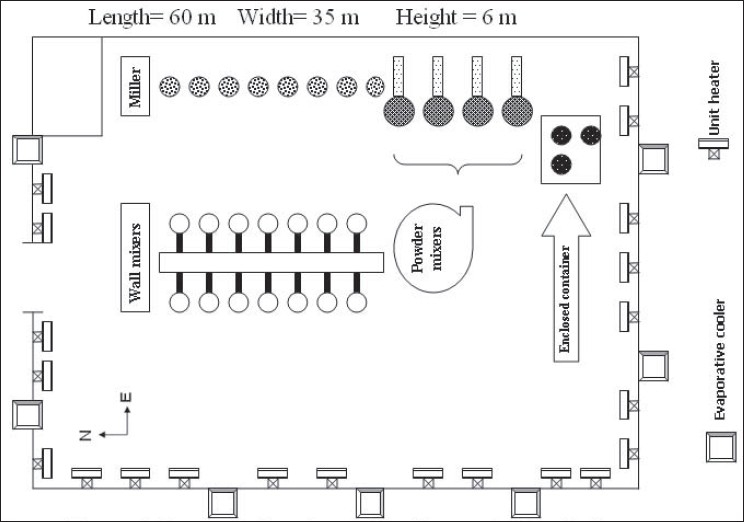
Layout of equipment in the production hall

The paint production process consisting of mixing, milling, and shearing (canning) stages were located in one hall. Figure 1 illustrates the layout of different types of mixers and mills used in this factory. Transportation and technical limitations would not let to partition the process. Natural ventilation was the only means of diluting air pollutants in the absence of mechanical ventilation. The lids of paint tanks that influence the ventilation capacity were not usable in all situations. Thus, workers were expected to experience high concentrations of evaporated solvents in their breathing zone. However, the application of personal protective equipments was limited for many reasons including their price, effectiveness, and application assurance.

American Conference of Governmental Industrial Hygienists (ACGIH) recommends dilution ventilation for paint mixing halls, where the mixing tanks are equipped with lids. According to its ventilation standard VS-75-30,[5] ACGIH suggests to exhaust polluted air from hall sides (close to mixing tanks) and supply fresh air from top center of the hall. Based on this ventilation standard, the pressure should be slightly negative in paint mixing hall. For this purpose, the exhausted air flow rate must be 5% more than supplied air flow rates (e.g., *Q*_exhaust_ = 1.05 *Q*_supply_). According to the same standard, supply and exhausted air flow rates are recommended to be 10-12 air change per hour.[5] When the paint tanks are without lids, ACGIH has no especial suggestion, but its ventilation standard number VS-70-20[5] is considered for solvent degreasing tanks that is the most similar process to the paint tanks and mixers without lids.

**Figure 2:**
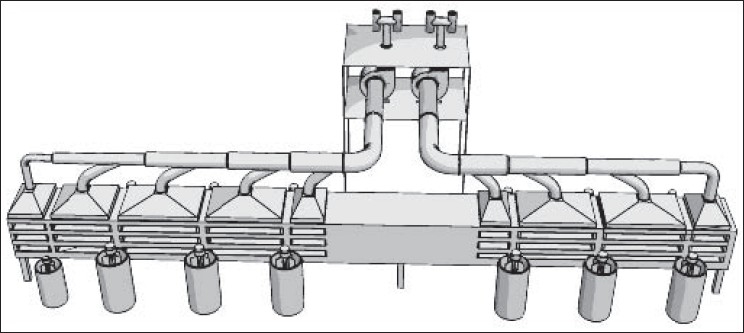
Local exhaust ventilation of wall mixers (slotted lateral hoods)

**Figure 3:**
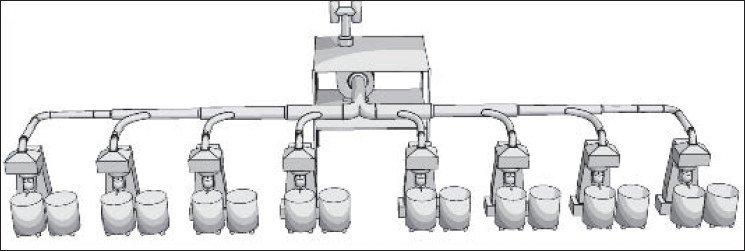
Local exhaust ventilation of Millers (eight canopy hoods near the pollution sources)

**Figure 4:**
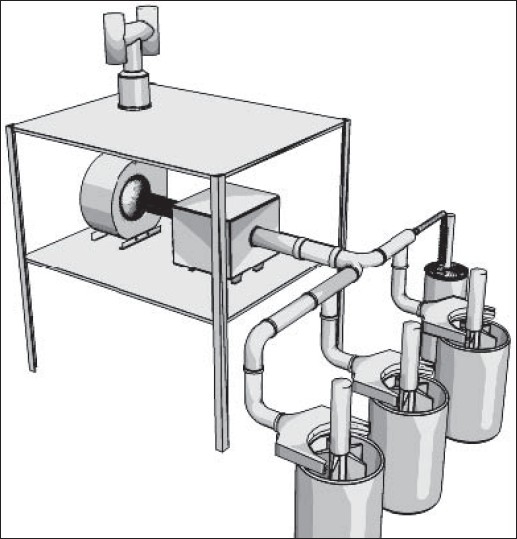
Local exhaust ventilation of powder mixers (includes four semi-circular laterals slotted hoods)

**Figure 5:**
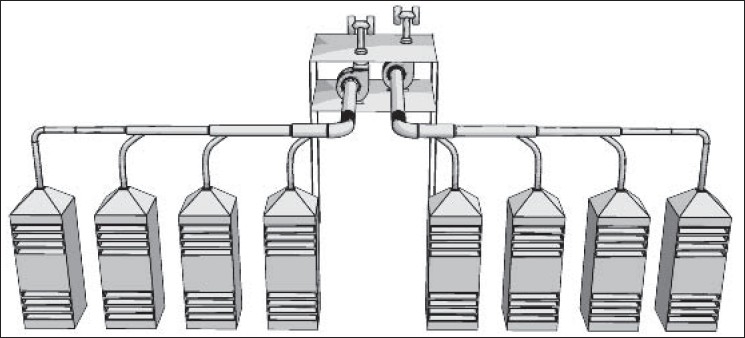
General exhaust ventilation systems (each system includes four lateral slotted hoods that exhaust air from floor and upper zones of the hall

**Figure 6:**
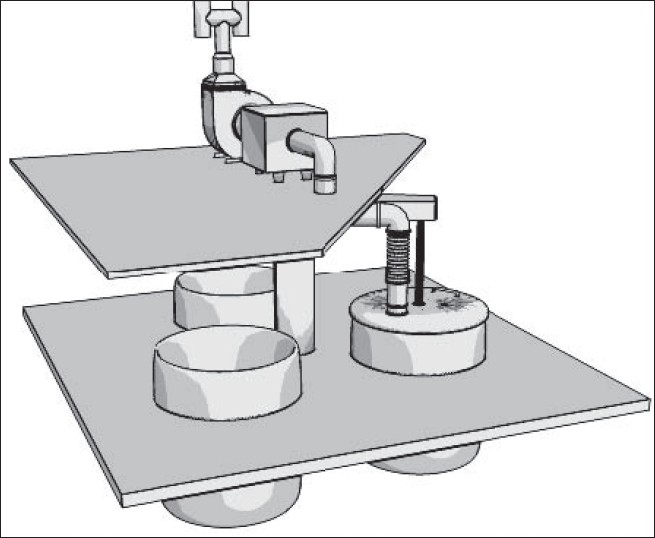
Exhaust ventilation of enclosed container (a single branch system attached to the container cap that can be assembled on each container in turn)

On the contrary, ACGIH does not recommend dilution ventilation for high toxic pollutants with threshold limit values (TLVs) less than 100 ppm (such as toluene and benzene). In these situations, local exhaust ventilation systems are highly recommended.[5] American Society of Heating, Refrigeration and Air-conditioning Engineers (ASHREA) and US Environmental Protection Agency (US EPA) emphasize on the most possible enclosure of paint manufacturing equipments. According to their literatures, local exhaust ventilation accompanying with dilution ventilation systems would act the best in controlling of pollutants in paint manufacturing processes. An atmosphere of flammable liquid safeguarded against fire and explosion in production halls usually will be kept below 25% of the lower explosive level (e.g., with a safety factor of 4).[5] This will fulfil US National Fire Protection Agency (US NFPA) and ACGIH recommendations on fire prevention of organic solvents in these areas as well.[5] In present study, recommendations of several knowledgeable institutes were taken into consideration. Because most of them were similar to each other, ACGIH recommendations were finally applied. For this purpose, a combination of local exhaust ventilation based on ventilation standard VS-70-20 and general dilution ventilation based on ventilation standard VS-75-30 both recommended by ACGIH were implemented.[5] Exhausted air was designed for 20% more than recommended values. As mentioned previously, 10-12 air change per hour (based on standard VS-75-30) and 50 cfm/ft of tank open area (based on standard VS-70-20) were considered as main design criteria. Seven local exhaust ventilation systems were designed and implemented according to ACGIH industrial ventilation standard handbook.[5] All parameters in ACGIH data sheet were formulated in Microsoft Excel program for detail design.[5] All pressure losses were calculated using velocity pressure method described in ACGIH ventilation handbook.[5] “Balanced by Design” method described in the same reference was applied to balance the static pressure of each branch with its main duct. However, several dampers (gates) were employed in some critical locations for balancing purposes.[5] The total supply air flow and consequently the total exhaust air flow were designed based on 10-12 air changes per hour according to ACGIH ventilation standard VS-75-30. Ducting was made of galvanized sheets with appropriate thicknesses for ducts, elbows, and hoods. Fiber dust collectors with 98% efficiency were selected for a few dry mixing processes. Fans were selected based on the calculation results. Figures 2-6 show 3D configuration of different ventilation systems applied to different processes.

### Personal air sampling and analytical methods

Toluene and xylene are the most common organic solvents in paint manufacturing factory under study. Thus, the vapors of these two substances were measured and analyzed with and without the application of implemented ventilation systems. For this purpose, 32 personal air samples were collected from breathing zone of workers using Occupational Safety and Health Administration (OSHA) analytical method No.12 before and after the application of ventilation systems. Few samples were shown invalid during their analysis so the post control samples decreased to 19.

All samples were collected by adsorption using charcoal tubes (coconut charcoal 20/40 mesh, 50/100 mg, SKC, USA), with a 100-mg sampling section and 50-mg back-up section according to OSHA No.12 analytical method.[6]

**Figure 7:**
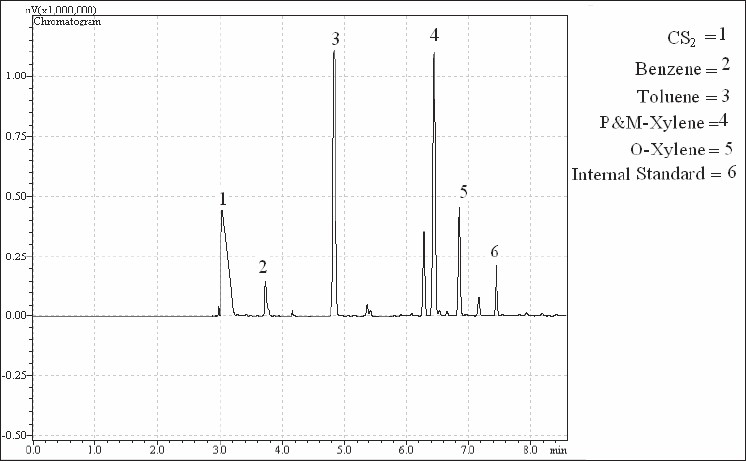
Chromatograph of one of the samples (part A of the charcoal tube)

**Figure 8:**
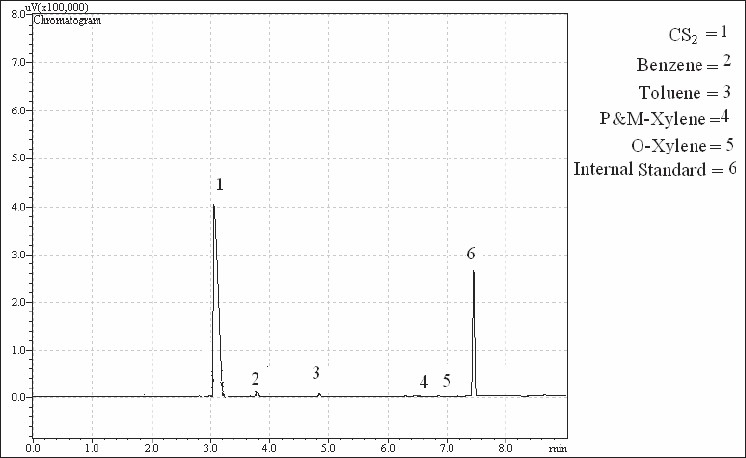
Chromatograph of one of the samples (part B of the charcoal tube)

Manufacturing lots were used with desorption efficiencies determined for each lot. SKC model 224-44EX pumps operated at approximately 50 ml/min were used for air sampling. All pumps were calibrated before each use by a calibrated rotameter. Sampled air and contaminant vapor volumes were corrected for density variation due to ambient temperatures and pressures changes from 760 mmHg and 25°C. The occurrence of breakthrough in samples taken for duration of 4 h and flow rate of 50 ml/min were examined. Then samples with breakthrough of more than 10% on the rear charcoal section were excluded from the study. The initial desorbing solvent for all samples was carbon disulfide. Both parts of samples (front and rear) were desorbed separately with carbon disulfide, taking 30 min time for desorption, and analyzed by gas chromatography (GC). Approximately 63 charcoal tube samples were analyzed in the study, including nine blanks (one per day per tube lot). In this study, Cumene (C9H12) was used as internal standard (IS) in order to remove the errors of sample preparation lost. Thus, 0.2 µl of Cumene was added to all vials during sample preparation. All chromatography was conducted on a SHIMATZU 175A series GC with a flame ionization detector. The resulted chromatograms were logged using GC Real Time Analysis software. Standard curves were created to quantify the samples [Figures 7 and 8].

## RESULTS

**Figure 9:**
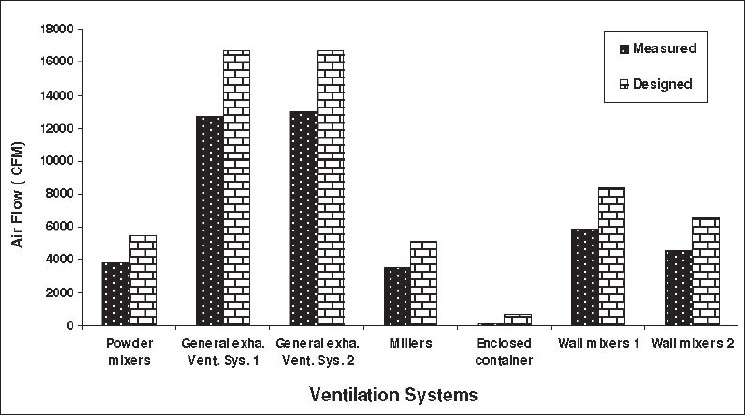
Comparison of designed air flow with measured air flow

The performance of the implemented ventilation systems was tested. For this purpose, exhaust flow rates (Q), total pressure (TP), static pressure (SP), and velocity pressure (VP) at different parts of each ventilation system were measured based on ACGIH and British Standard (BS) recommendations. Pitot tubes, monometers, and anemometers from Air Flow Company (UK) were used to measure the aerodynamic parameters. Air density was corrected for barometric pressure, temperature, and static pressure where it was essential. Measuring equipments were calibrated by their manufacturer's local representative prior to their use in the study. In Figure 9, the design flow rates are compared with measured flow rates. This figure shows that apparently the installed system is not working as designed system. In fact the designed parameters have not been completely established according to ventilation detail design. These results show that 60-70% of the designed flow rates were achieved in this project. Unfortunately, the local fan manufacturers are not able to deliver the exact required fans. However, the difference between design flow rate and measured flow rate are mainly due to this fact and measuring discrepancy. However, 20% over design was considered during system design, so this difference between designed parameters and real achievements has little impact on final results.

Benzene, toluene, P and M-xylene, and O-xylene were detected from personal air samples chromatograms. In Table 1, the concentrations of detected pollutants before and after application of ventilation system are listed. In this table, the concentrations of para and meta-xylene are mentioned in a single value, because their analytical peak in GC set were simultaneously appeared. However, there need only total xylene value for assessment as well. The mean values of benzene, toluene, and total xylene concentrations before applying engineering controls were 31.98, 105.82, and 145.16 ppm, respectively. The results showed that these values were reduced respectively to 4.5, 44.5, and 97.73 ppm after the application of ventilation systems. The independent *t*-test before and after ventilation systems activation was done for benzene, toluene, and xylenes. These results showed that the reduction of these pollutants were statistically significant (*P* < 0.001; Table 1).

**Table 1:** Result of independent t-test (all values in ppm)

Pollutant (ppm)	Before control			After control			MD	t	Significant (two-tailed)
					
	n	Mean	SD	n	Mean	SD			
Benzene	32	31.98	26.08	19	4.50	3.64	27.50	5.84	0.001
Toluene	32	105.82	74.88	19	44.50	24.39	61.33	4.27	0.001
P and M-xylene	32	145.16	39.70	19	56.16	30.52	89	8.40	0.001
O-xylene	32	76.60	28.83	19	41.57	26.56	35.03	4.32	0.001

MD - mean difference, SD - standard deviation.

**Figure 10:**
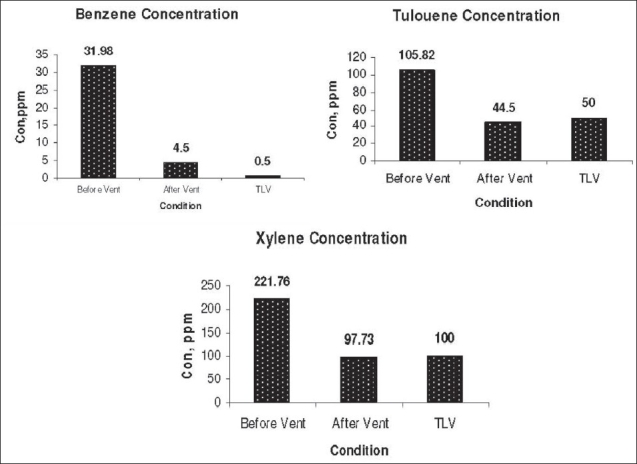
The comparison of concentrations with TLVs (all values in ppm) early benzene

The measured concentrations of pollutants were compared with ACGIH recommended TLVs [Figure 10]. The results show that the ventilation system was able to control the major pollutants of toluene and xylene, but it failed to control benzene.

## DISCUSSION

Existence of benzene in benzene-free solvents used in paint-producing factories can be considered as a warning sign. Benzene was usually contained as impurity in paint, thinner, or solvent until early 1990s, even in low values of less than 1%.[7] Bang *et al*. reported in 1996 that ambient benzene levels in painters and printing work places were 0.31 ppm (0.02-3.26 ppm) and 0.25 ppm (0.02-3.95 ppm), respectively.[8] The amount of benzene as impurity in thinners has decreased since 1980s. Paik *et al*. analyzed 108 different thinners in 1998. Most of the thinners were benzene free, but eight still contained benzene as impurity.[9] Lee *et al*. has analyzed 70 different thinners used in automobile manufacturing factory in 2002. He found that seven thinners contained benzene, but the amount of benzene was less than 0.1%.[10]

The application of solvents in uncontrolled workplaces with high exposure levels was found to be a potent of bone marrow toxicant. However, it was not until 1970s that epidemiological studies conducted in the USA discovered excess of acute and chronic leukemia. This was essentially the starting point of many more detailed epidemiological investigations that examined leukemia in well-defined cohorts with estimated exposure profiles. These investigations enabled quantitative risk assessments to be developed.[7] The common type of leukemia caused by benzene was acute non-lymphatic leukemia.[11,12] However, any type of hematopoietic disease can be developed because benzene toxicity affects the proliferating process of the stem cell.[7,13] The complete removal of benzene from workplace is ideal, but it is difficult to achieve in paint production process using present ACGIH ventilation standards. Benzene was not supposed to be existed in raw materials in this factory, so it may be leaked from purification processes of refineries. Therefore, the VS recommended by ACGIH would be significant if basic solvents for paint manufacturing could be benzene-free. However, in regard to benzene exposure, mere conventional VS systems could not safeguard workers’ health. Therefore, using better quality solvents with lower content of benzene are strongly recommended. For time being, suitable personal protective equipment and routine health surveillance, such as biological monitoring of benzene, should be considered for exposed workers.[14]

## References

[1] Ridgwaya P, Nixon TE, Leach JP (2003). Occupational exposure to organic solvents and long-term nervous system damage detectable by brain imaging, neurophysiology or histopathology. Food Chem Toxicol.

[2] (1977). NIOSH, Occupational Diseases - A Guide to their Recognition, in Publication No 77-181.

[3] Williams PR, Knutsen JS, Atkinson C, Madl AK, Paustenbach DJ (2007). Airborne concentrations of benzene associated with the historical use of some formulations of liquid wrench. J Occup Environ Hyg.

[4] McMinn BW (1992). Control of VOC emissions from ink and paint manufacturing processes. C.T. Center. Environmental Protection Agency.

[5] (1995). ACGIH, Industrial Ventilation a manual of recommended practice.

[6] Elskamp CJ OSHA Sampling and Analytical Methods, Method no: 12, September 1979, August 1980, Revised, Organic Methods Evaluation Branch OSHA Analytical Laboratory.

[7] Kang SK, Lee MY, Kim TK, Lee JO, Ahn YS (2005). Occupational exposure to benzene in South Korea. Chem Biol Interact.

[8] Bang SH, Kim KJ, Yum YT (1996). Urinary S-phenylmercapturic acid as a biomarker for biological monitoring in workers exposed to benzene. J Korean Soc Occup Environ Hyg.

[9] Paik NW, Yoon CS, Zoh KE, Chung HM (1998). A study of component of thinners using in Korea. J Korean Soc Occup Environ Hyg.

[10] Lee KS, Kwon HW, Han IS, Yu IJ, Lee YM (2003). A study on the reliability of material safety data sheets for paint thinner. J Korean Soc Occup Environ Hyg.

[11] Swaen GM, Meijers JM (1989). Risk assessment of leukemia and occupational exposure to benzene. Br J Ind Med.

[12] Aksoy M, Erdem S, Dincol G (1976). Types of leukemia in chronic benzene poisoning: A study in thirty-four patients. Acta Haematol.

[13] Agency U.S.E.P. (1997). Carcinogenic Effects of Benzene: An Update. National Center for Environmental Assessment Office of Research and Development.

[14] (2005). ACGIH, Recommended Threshold Limit Values for Work Environment. A.C.O.G.I. Hygienists, editor.

